# Supplementary material for: Cross-Protective Immune Responses Induced by Sequential Influenza Virus Infection and by Sequential Vaccination With Inactivated Influenza Vaccines
Source: Front Immunol. 2018 Oct 9;9:2312. doi: 10.3389/fimmu.2018.02312 (PMC6189474; doi:10.3389/fimmu.2018.02312)
Supplement: Supplementary file 1 [file Data_Sheet_1.docx]

***Supplementary material***

Wei Dong^a^, Yoshita Bhide^a^, Federica Sicca^a^, Tjarko Meijerhof^a^, Kate Guilfoyle^b*^, Othmar G. Engelhardt^b^, Louis Boon^c^, Cornelis A.M. de Haan^d^, George Carnell^e^, Nigel Temperton^e^, Jacqueline de Vries-Idema^a^, David Kelvin^f,g^, Anke Huckriede^a#^

#Address correspondence to Anke Huckriede [a.l.w.huckriede@umcg.nl](mailto:a.l.w.huckriede@umcg.nl)


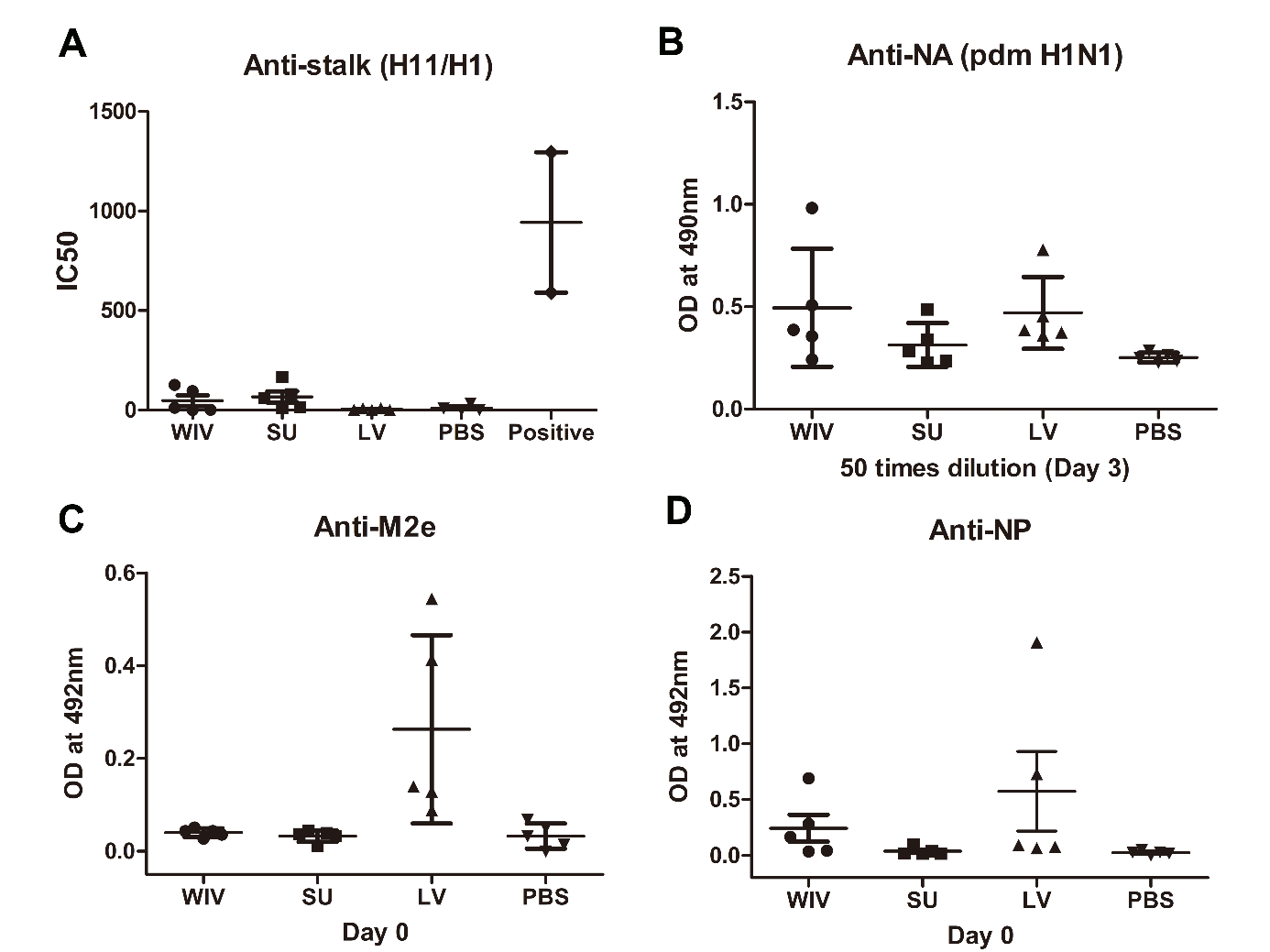


**Supplementary Fig 1. Cross-reactive antibodies against conserved proteins/protein domains induced by sequential infection or immunization.** Mice were primed and boosted as described in the legend to Fig 1. Serum samples were collected 28 days post boost (day 0; A, C, D) or 3 days post-challenge (day 3; B). (A) Antibodies against A(H1N1)pdm09 HA stem region were determined by pseudotype HA stalk neutralization assay as described in M&M. Antibodies against A(H1N1)pdm09 (B), M2e (C) and NP (D) were determined by ELISA. Individual data, mean and SEM are depicted.


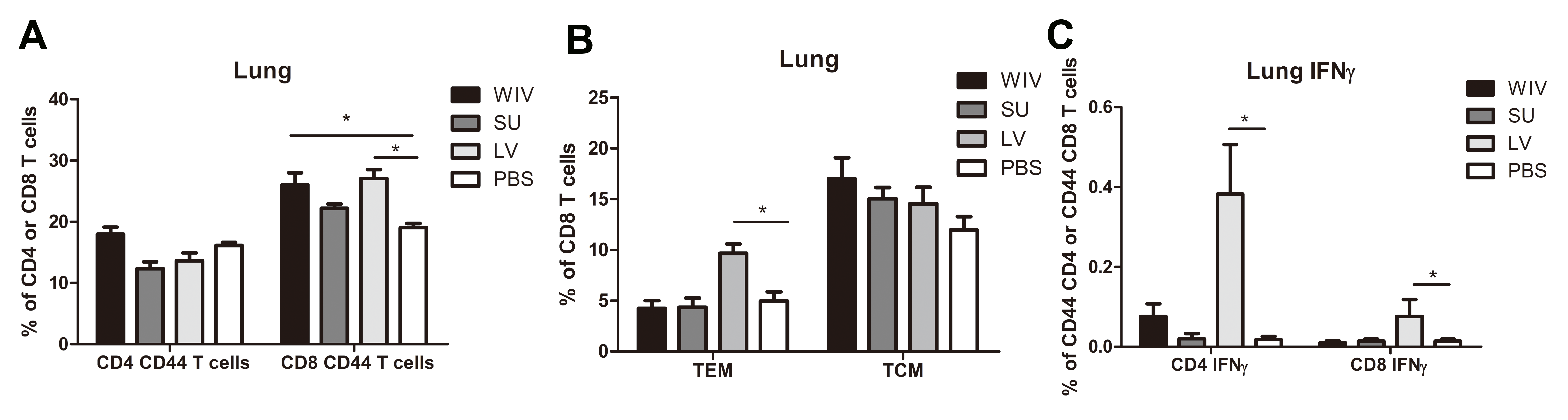


**Supplementary Fig 2. T cell immune responses induced by sequential infection or immunization in lung.** Lung lymphocytes harvested on day 28 post the second infection/vaccination, were rested overnight and then stained for CD4, CD8, CD62L and CD44. (A) CD4+CD44+ and CD8+CD44+ memory T cells in lung were determined by FACS. (B) CD8+CD44+CD62L- effector memory T cells (TEM) and CD8+CD44+CD62L+ central memory T cells (TCM) in lung were analyzed by FACS. For determination of influenza-specific T cells, lung lymphocytes were stimulated overnight with A(H1N1)pdm09 WIV and anti-CD28 overnight in presence of protein transport inhibitor. (C) Percentages of IFNγ-producing cells among CD4+CD44+ and CD8+CD44+ T cells in lung were analyzed by flow cytometry. Data represents mean values + SEM, *, p<0.05, Mann-Whitney U test.
